# Making sense of the economics of abortion in the United States

**DOI:** 10.1111/psrh.12288

**Published:** 2024-11-13

**Authors:** Tracy A. Weitz

**Affiliations:** ^1^ Department of Sociology and the Center on Health, Risk, and Society American University Washington DC USA

## Abstract

In 2023 the editors of *Perspectives on Sexual and Reproductive Health* issued a special call for papers related to the economics of abortion. Ten of those submissions are included in this volume and address critical issues including: (1) the role Medicaid continues to play in abortion access and how changes in state Medicaid coverage of abortion have expanded and restricted abortion care use; (2) how low‐income individuals without insurance coverage for abortion utilize resources from abortion funds and through crowdsourcing platforms; (3) how the price of medication abortion has decreased with the availability of telemedicine medication abortion and how providers of that service are making efforts to reduce those prices even further; and (4) how legally restricting abortion access has significant economic implications for state economies and the US society as a whole. In this introduction, I review the general scope of prior research on the economics of abortion in the US as it relates to stigma‐induced silences, abortion seekers, abortion providers, and abortion assistance organizations. I then highlight the new contributions made by the articles contained in this special issue.

## INTRODUCTION

Abortion is an issue of money, as economics influence the need for abortions, abortion seekers' ability to access abortions, efforts to provide abortions, and the consequences to individuals and society of people not obtaining wanted abortions. In June 2022, the United States (US) Supreme Court eliminated national legal protection for abortion in its *Dobbs v. Jackson Women's Health Organization* (*Dobbs*) decision that overturned the 1973 *Roe v. Wade* (*Roe*) decision establishing a federal right to an abortion through the second trimester of pregnancy.^1^ Consequently, states can now establish their abortion laws and in 2024, one in three women of reproductive age lived in states where all or most abortions were banned.^1^ These bans have exacerbated the economic challenges of providing and accessing abortion in the US as well as raised broad economic implications for the states with such policies. Unfortunately, many abortion access and provision challenges existed prior to *Dobbs* 2, 3, 4 and remain relevant even in states that did not further restrict abortion following the *Dobbs* decision, for example, see references 5, 6, 7, 8, 9, 10. Thus, understanding the current economic issues related to abortion in the US requires examining both the impact of *Dobbs* as well as how the system operates where abortion is broadly legal.^2^

To produce a more comprehensive and multidisciplinary understanding of the situation in the US, in 2023 the editors of *Perspectives on Sexual and Reproductive Health* issued a special call for papers related to the economics of abortion. Ten of those submissions are included in this volume and address critical issues including: (1) the role Medicaid continues to play in abortion access and how changes in state Medicaid coverage of abortion have expanded and restricted abortion care use; (2) how individuals living on low incomes without insurance coverage for abortion utilize resources from abortion funds and through crowdsourcing platforms; (3) how the price of medication abortion has decreased with the availability of telemedicine medication abortion and how providers of that service are making efforts to reduce those prices even further; and (4) how legally restricting abortion access has significant economic implications for state economies and US society as a whole.

In this introduction, I review the general scope of prior research on the economics of abortion in the US as it relates to stigma‐induced silences, abortion seekers, abortion providers, and abortion assistance organizations. I then highlight the new contributions made by the articles contained in this special issue. While there is a large body of already published research on the impact of legal abortion on US society, I have chosen not to specifically summarize this research in this introduction as the *Brief of Amici Curiae economists in support of respondents in Dobbs v. Jackson Women's Health Organization* reprinted in this special issue does an excellent job.

## BACKGROUND AND CONTEXT

### Abortion, money, and stigma

There is a unique form of abortion stigma tied to the role of money.^11^ Due to the criminal nature of abortion prior to *Roe*, individuals were able to extract high financial payments for illegal services.^12^, ^13^, ^14^ There were also legitimate physicians who performed abortions prior to *Roe*,^15^ but the descriptions of the pre‐Roe providers are usually of those who financially profited. The reality and the taint of the “abortionist as profiteer” played out in the earliest days of legalization post*‐Roe*. Goldstein identified that profit seeking and a commitment to women's equality were two factors that drove entrepreneurial physicians to establish abortion practices immediately after *Roe* ^16^ and debates over whether abortion providers should be for‐profit or not‐for‐profit resulted in one of the first disagreements within the abortion rights social movement.^17^ Reflecting this tension was the experience of Roy Lucas, the lawyer who wrote the initial *Roe* arguments. In addition to his work on the early abortion legalization cases, Lucas also represented for‐profit abortion referral services that challenged a New York law prohibiting their existence. Because he supported these for‐profit entities, he was not allowed to serve on the board of directors for the National Association for the Repeal of Abortion Laws (NARAL).^3^, ^17^ His support was seen as inconsistent with the mainstream pro‐choice community's position against garnering profit from abortion care.

Opponents of abortion rights have mobilized the pro‐choice movement's longstanding discomfort with the relationship between abortion and money to disparage all providers who offer abortion care.^18^ They refer to abortion providers as the “abortion industry” and accuse them of being driven entirely by profit.^19^, ^20^, ^21^, ^22^ Anti‐abortion rights activists leverage testimonials by former employees to prove the role of profit in abortion provision.^23^ As I discuss later in this article, there are business realities associated with providing abortion care that are often misunderstood, and thus abortion providers are often reluctant to discuss these issues publicly. The result is that the reproductive health, rights, and justice movement^4^ is generally uncomfortable discussing abortion and money.^11^

Abortion stigma also attaches uniquely to money in public opinion polling. In 2018, a research team at New York University updated the abortion questions in the General Social Survey (GSS), a scientifically rigorous public opinion survey conducted since 1972 on various social issues. Testing whether there were alternatives to simply asking about the legality and morality of abortion, the new abortion questions asked people about the kinds of help they would extend if a close family member or friend decided to have an abortion.^24^ One option was paying for the abortion. Although over 90% of people said they would offer emotional support and nearly three‐quarters would help with arrangements like a ride or childcare, less than half would help pay for the abortion itself.

### Economics and abortion seekers

#### Economics and the need for abortion

The US abortion patient population reflects long‐standing health and economic inequities.^25^ When last measured in 2021–2022, almost three‐quarters of all abortion patients were living at or below 200% of the federal poverty level (FPL) and the majority were women and other pregnancy capable people of color.^26^ While contemporary research has moved away from asking abortion seekers the reasons why they want or need an abortion, decades of prior literature found that many people identified economic issues as the reason for their abortion.^27^, ^28^ In a nationally representative study of US abortion patients conducted in 2004 over 70% of people selected “I can't afford a baby right now” as a specific reason for the decision to have an abortion.^29^ In the Turnaway study, which measured the consequences of obtaining and being denied a wanted abortion, Foster et al. found that 40% of respondents expressed “not financially prepared” as a main reason for having an abortion; other factors such as “interferes with future opportunity,” expressed by another 20% have financial underpinnings as well.^30^

#### Economics and the ability to obtain the abortion

The US is the only high‐income country in the world without a national healthcare system for its citizens.^31^ Instead, private insurance is often extended as a benefit of employment and public health insurance is available to some low‐income, disabled, and elderly individuals. Uninsured individuals are financially responsible for paying for their care. Abortion patients are more likely to have public insurance or be uninsured than the general population.^32^ However, many of these individuals are prohibited by law from using their public insurance to pay for abortion care. Since 1976, the federal government has imposed the Hyde Amendment (Hyde), which prohibits federal funds from paying for most abortions.^33^ Although Hyde allows for coverage of abortions for pregnancies resulting from rape or incest or when a pregnant person's life is in danger, an investigation conducted by the US Government Accounting Office (GAO) found poor compliance.^34^

Under Hyde, state governments are free to use their own funds to cover abortion care, and indeed 19 states do so.^5^, 33, 35 Research has examined the consequences of states covering abortion under the public insurance program known as Medicaid for those living on low incomes. Using data from a population‐based survey of women of reproductive age in six states, Jackson and Rendall recently found that Medicaid coverage of abortion care was associated with a 37 percentage‐point higher cumulative lifetime abortion incidence among Medicaid‐insured women relative to women not insured by Medicaid for the state where Medicaid covered abortion.^36^ In a study of Medicaid expansion in Oregon after promulgation of the Affordable Care Act, Harvey et al. found that by increasing the number of people covered, Medicaid expansion had a measurable impact on the gestational timing of abortions as more abortion seekers were able to access care at an earlier point in pregnancy.^37^ Caution should be used in universalizing from the Oregon experience, as studies in other states have found that trying to obtain Medicaid coverage can cause a delay in obtaining care.^38^, ^39^, ^40^, ^41^

Because most abortion seekers are living on low incomes and cannot use their insurance for care the most consistent finding in research with US abortion seekers is the preeminence of raising the money as a barrier to care and a psychological stressor.^40^, ^42^, ^43^, ^44^, ^45^, ^46^, ^47^, ^48^ Studies report that in order to cover out‐of‐pocket costs related to paying for and accessing abortion care, women and other pregnancy capable people defer paying for rent, utilities, food, or child‐rearing expenses.^30^, ^42^, ^49^, ^50^, ^51^ Further, lack of abortion affordability was identified as a leading factor in why pregnant people seek to end their pregnancies outside the formal US health care system (often referred to as self‐managed abortion).^52^, ^53^

A few studies have specifically examined the price of abortion. Upadhyay et al. reported 2020 average prices of medication abortion at $560, first‐trimester instrumentation abortion at $575, and second‐trimester instrumentation abortion at $895.^54^ The Guttmacher Institute's 2017 Abortion Provider Census found that the median amount charged by clinics for an abortion at 20 weeks gestation was $1670 (range $410–$5386).^55^ There are no published studies of the price of abortion after 20 weeks. However, media stories of abortions in the third trimester suggest the price may be tenfold higher than that reported in the Upadhyay publication.^56^, ^57^, ^58^ Regardless of whether they are seeking care earlier or later in pregnancy, the price of abortion care is unaffordable for most low‐income abortion seekers. Thus, abortion can be conceptualized as a catastrophic health expenditure for many people.^59^ Consequently, economists suggest that the price of abortion impacts overall utilization.^60^ In 2008, Medoff concluded that for the period 1982–2000 approximately 20% of the decline in the incidence of abortion was due solely to the increase in the real price of obtaining one.^61^

Transportation, lodging for an overnight stay, and childcare are also significant expenses for people who have to travel for abortion care.^62^, ^63^, ^64^, ^65^, ^66^, ^67^, ^68^, ^69^, ^70^, ^71^, ^72^, ^73^ Reflecting that burden, researchers have found that greater distance from a facility negatively impacts a community's abortion rate.^68^, ^74^ These findings have been demonstrated in local empirical studies with abortion patients in Texas, Alabama, and Wisconsin^69^, ^75^, ^76^ as well as with abortion seekers online.^77^

Studies spanning two decades found that when Medicaid does not cover abortions, approximately one‐quarter of Medicaid‐eligible pregnant people do not obtain a desired abortion.^78^, ^79^ A recent study of people seeking abortion care information online found that people living in states that do not provide Medicaid coverage have significantly higher odds of still seeking an abortion 4 weeks later than individuals living in covered states.^46^

Timing of obtaining abortion care is important for several reasons, including a small increased medical risk associated with abortions at more advanced gestations,^80^ yet still always less risky than a full‐term delivery.^81^ Delays also matter because the price of abortion increases after the end of the first trimester, resulting in a phenomenon known as “chasing the fee,” when someone finally gets the money together for the abortion, but the price of the abortion has increased due to the delay, and now they again do not have enough money. Delays ultimately matter because they can result in never obtaining the desired abortion. In the Turnaway study, almost 60% of those denied an abortion for arriving after the clinic's gestational limit reported that the reasons for delay included travel and procedure costs.^82^

The economic consequences of not obtaining a desired abortion are significant. Miller et al. found that being denied a wanted abortion has large and persistent negative effects on measures of financial well‐being.^83^ Taking advantage of the success of the Turnaway Study in recruiting a hard‐to‐reach population, the authors link the study participants to 10 years of credit report data. From this, they examine aspects of financial stress such as lateness in paying bills, having bills sent to collection agencies, and serious adverse financial events like evictions and bankruptcies. They also assess markers of financial self‐sufficiency and resiliency, such as having access to a reserve of credit, and information on consumer borrowing in the form of credit cards, mortgages, and automobile loans. The authors find that outcomes evolved similarly for those who obtained and were denied abortions prior to the abortion encounter. Following the encounter, women who were denied an abortion experienced a large increase in financial distress that remained for several years including increased bancrupcies. Women who were unable to obtain a wanted abortion were also more likely to later rely on governmental supports compared to women who received a wanted abortion.^84^

### Economics and abortion providers

#### Economics and marginalization from mainstream medicine

As a result of market forces, technological advancements, state regulation, anti‐abortion violence and harassment, and social movement advocacy, abortion as a health service is offered outside mainstream medicine in the US.^85^, ^86^ Consequently, since *Roe*, over 90% of all abortions have been performed in specialty outpatient clinics known colloquially as “abortion providers.”^87^, ^88^ In 2020, only 4% of all abortions were performed outside those facilities.^89^ These abortion providers are either part of the Planned Parenthood system or what is generically called an “independent abortion provider” (or indy for short).

Prior to *Dobbs* there were only 800 of these publicly identifiable abortion providers,^90^ a number that had been shrinking over the previous two decades. Almost all these facilities were located in urban environments. The Guttmacher Institute Abortion Provider Survey has documented this consolidation by measuring the percentage of women living in a county without an abortion provider, which rose from 32% in 1996 to 38% in 2020.^89^, ^91^ Since *Dobbs*, the number of facilities offering abortions has changed slightly with 63 providers no longer performing abortions in states with total abortion bans and 21 new providers opening in states without total bans.^92^ Despite these shifts in care site provision, the number of abortions performed in the formal health care system has been increasing since *Dobbs*.^93^ The reasons for this change have not yet been scientifically documented. However, researchers posit that pre‐existing upward trends in abortion use, greater availability of telemedicine services, more financial resources to help abortion seekers pay for and reach care, and favorable changes in state laws protecting abortion access, have all contributed to the higher number of abortions.^94^

#### The economics of providing care

Anti‐abortion hostility and increasingly restrictive regulations are reasonably blamed for the reduction in abortion providers before *Dobbs*.^71^, ^95^, ^96^, ^97^ But so too was a declining demand for abortion services. Because most abortion providers are not part of larger healthcare systems,^6^ their financial security is tied predominately to reimbursements for the specific services they provide. To make the math work, abortion providers rely on distributing fixed costs (e.g., rent/mortgage, salaries, and equipment) across the number of abortions performed. In other words, the more abortions, the better the financial picture.

When the alarm bells rang regarding the “greying of the abortion provider” in the 1990s,^98^, ^99^ there were 1.6 million abortions performed annually in the US.^100^ This number declined to approximately 860,000 in 2017,^87^ and as such, the existing providers were serving fewer patients per facility, making their financial sustainability tenuous. Despite this reality, few research studies examined the business decisions of abortion providers. Instead, the media reported these changes.^101^ For example, in 2000, the *New York Times* published an article aptly titled “As Abortion Rate Decreases, Clinics Compete for Patients,” in which clinic owners discuss how competition was responsible for the concentration of clinics in urban areas and the downward pressure on the price for services.^102^

Abortion providers experienced another squeeze on their business model with the rise in telemedicine medication abortion. In 2020, in response to the need to reduce the risk of face‐to‐face transmission of COVID‐19, the US Food and Drug Administration (FDA) temporarily allowed the mailing of medication abortion drugs.^103^ As a result of the change, new abortion providers that offer only medication abortion via telemedicine entered the market, often financed with venture capital.^104^, ^105^ By June 2023, approximately 7000 patients a month were obtaining care through one of these new virtual‐only medication abortion providers.^106^ The result was even fewer abortions per brick‐and‐mortar facility.^107^

This FDA change to the in‐person dispensing requirement was made permanent in 2021. Since then, telemedicine medication abortion care has continued to expand despite initially only being offered to people receiving care in the 22 states and the District of Columbia where telemedicine abortion care was legally permitted.^108^ Following the *Dobbs* decision, legislatures in a few progressive states passed laws to protect healthcare providers wanting to serve telemedicine patients residing in abortion‐ban states. Eight states now have such laws: Massachusetts, Washington, Colorado, Vermont, New York, California, Maine, and Rhode Island.^109^ While the individual state laws differ, practically the laws mean that clinicians practicing in these states have legal protections to consult with and mail medications to patients who reside in states where this service is prohibited. The result is that many people living in states where abortion is illegal or criminalized are accessing care via telemedicine. The data tracking project #WeCount of the Society of Family Planning documented that as of June 2024, almost 20% of all US abortions were performed via telemedicine medication abortion.^93^ This is great for abortion seekers but may be challenging the financial viability of some abortion providers.

#### The economics of pricing abortion care

As discussed earlier, most abortion seekers are living on low incomes, and most must pay out‐of‐pocket or rely on Medicaid coverage if it is allowed in their state. Both payor options have significant implications for how much abortion providers can get paid for their services. Unlike other healthcare services, the price of abortion has stayed relatively stable over time^87^, ^110^, ^111^ in part because abortion providers have tried to keep the price low for abortion seekers who are overwhelmingly low‐income and because raising prices means people will not be able to access care.

What it actually costs to provide different types of abortion care in different service delivery models has received minimal attention. In 2007, Afable‐Munsuz et al. published the results of a study of 11 clinics delivering medication abortion care.^112^ The models of care in this study involved multiple visits and had wide variation in time spent in specific aspects of care, including patient counseling and follow‐up visits. Despite this variation, patient satisfaction was high across all practices. The total episode cost for providing medication abortion ranged from $252 to $460. Today, the average cost of provision might be lower, given fewer steps in the medication abortion process and a reduction in the cost of mifepristone, or higher, due to increased staffing wages or facility costs. To answer this question, in 2018, Resources for Abortion Delivery engaged the same consulting firm used in the Afable‐Munsuz study to conduct a broader cost assessment of abortion provision as part of its work to support independent abortion providers. Overall, the study concluded that “a facility that provides abortion care at lower costs generally: (a) has moderate to high annual abortion volume, (b) is a specialty provider where at least 90% of all services provided are related to abortion care, and (c) has minimal overhead/ management expenses that, when distributed over the abortion service volume, do not significantly contribute to the total cost to provide abortion care.”^113^ ^,p.6^ This finding echoes prior data from the Guttmacher Institute that showed a relationship between volume and price. In 2017, nonspecialized clinics charged more than specialized clinics for both first‐trimester instrumentation and medication abortion.^55^ These findings are in line with older studies, which also found that large abortion providers are better able to compete and survive in the market.^114^

#### The economics of accepting insurance

Abortion providers make independent decisions as to whether to accept public and private insurance. One study found that due to administrative burdens, many providers do not accept insurance for abortion services,^115^ and a more recent study documented that the overall rate of acceptance is declining.^54^ Beyond this, there is no research on how private insurance reimbursement impacts abortion providers.

In contrast, there is a sizable, although somewhat old, body of literature on the impact of Medicaid acceptance on abortion providers. This research found that in states that do allow Medicaid coverage, cumbersome systems and low reimbursement rates threatened abortion providers' financial sustainability.^115^, ^116^, ^117^, ^118^, ^119^ Further, although federal Medicaid should cover care for abortions allowable under the Hyde Amendment, prior negative experiences attempting to obtain payment for Hyde‐allowable abortions led most providers in states without state Medicaid to stop seeking reimbursement.^119^

Notoriously a poor payor for health care, state Medicaid reimbursement rates for abortion services fall well below the total cost of providing care.^120^ Even if reimbursements do not meet the cost of providing a service, healthcare providers who accept Medicaid may not collect additional payment from Medicaid patients, except for cost‐sharing authorized under federal and state law. The implications for low reimbursement rates are evident when examining the scope of Medicaid's role as a payor of health services. In 2017, over half of all patients obtaining abortions in states with Medicaid coverage for abortion have the procedure covered by Medicaid.^32^

Only one study has systematically examined Medicaid reimbursement rates for first‐ and second‐trimester abortion care across the US. To contextualize those rates, Young et al. also compared Medicaid reimbursement rates for abortion care with an almost identical medical procedure: miscarriage management (spontaneous abortion) in the first and second trimesters of pregnancy. They concluded that Medicaid reimbursement rates for abortion are low, with median rates for first‐ and second‐trimester abortions covering approximately 37% and 41% of the actual price of the abortion. Further, they found that while induced abortion procedures are similar to miscarriage management procedures, Medicaid reimbursement rates for all abortion procedures were lower.^117^

Recently, KFF (formerly the Kaiser Family Foundation) released a report on the amounts reimbursed by state Medicaid programs for abortion care.^121^ It found significant variation across states and notably low reimbursement rates for abortions after the first trimester. The report also exposed that there are many unknowns about how abortion services are billed and reimbursed. For example, little is known about how states set their rates or what it takes to advocate for change. The recent experience in Illinois offers some lessons. At the time of the passage of House Bill 40 (HB40), Illinois reimbursed enrolled providers only $200 regardless of the gestational age of the pregnancy or the complexity of the abortion.^118^, ^122^ A state coalition of advocates, abortion providers, and Medicaid expert consultants successfully worked to develop a state system for Medicaid reimbursement that included rates at least comparable to current prices charged to clients paying out‐of‐pocket. Whether those amounts are sufficient to ensure the financial solvency of providers remains to be determined.

The National Health Law Program has documented the many ways in which telehealth/telemedicine reimbursement policies under state Medicaid programs limit access to abortion.^123^, ^124^ In some states, healthcare providers must have a preexisting relationship with a patient to bill for telehealth services. However, because abortion care is episodic, most abortion providers do not have preexisting relationships with abortion seekers. Another example of a limiting state requirement is that only physicians can bill Medicaid for telemedicine services. However, states with Medicaid coverage for abortion care usually also allow advanced practice registered nurses and physician assistants to provide that care; these clinicians often provide the majority of medication abortions.^125^ Due to existing telehealth policy and reimbursement barriers, Medicaid recipients find themselves unable to use their insurance for this service despite it being supposedly covered. No studies to date have examined the impact of these differential policies on overall abortion utilization or access equity.

### Economics and abortion assistance organizations

Whether due to lack of insurance, the inability to use insurance to cover abortion care, or the need to pay for expenses associated with reaching abortion care, abortion seekers living on low incomes and abortion providers turn to a complex web of non‐profit organizations. These organizations offer both financial assistance for the abortion procedure expenses and practical support to access the abortion service, including transportation, lodging, childcare, and emotional support. Already under‐resourced for the need, this web of organizations became even more critical after *Dobbs*. The costs associated with new travel burdens often accompany higher costs for abortion procedures later in pregnancy, which result from delays associated with navigating care outside a person's home state. Post *Dobbs*, three large national abortion assistance organizations and over 100 local or regional organizations comprise this financial and practical support network. There are no comprehensive data on the collective resources these entities dispense.

The most significant assistance organization, the National Abortion Federation (NAF) Hotline,^7^ helps cover the price of an abortion obtained from an independent abortion provider and offers some limited financial assistance for patients who must travel out of state. Internal Revenue Service (IRS) tax records for 2022 show that the NAF Hotline provided over $50 million in supports to individuals.^126^ A news story reported that for the first 6 months of 2024, the NAF Hotline partially funded over 60,000 patients for around 6 million dollars per month. In December 2023, the NAF Hotline released some limited information on travel supports showing an increase from zero hotel rooms and transportation tickets supported in June 2021 to over 200 combined supports for November 2023.^127^ To date, there are no studies of the impact of the NAF Hotline supports. The Planned Parenthood system can internally provide financial support to abortion seekers because of philanthropic giving to either the national organization or to one of its 49 affiliates. There are no effective means of tracking the resources provided to patients in this way. However, like the NAF Hotline, news reports suggest that the demand exceeds the organizations' available funds.^128^ Equally opaque was the third national abortion support organization known as “The Abortion Fund” (TAF), which operated out of the fiscal sponsor the Hopewell Fund. This fund was sunsetted in the summer of 2024 and there are no records of how many funds were made available to cover abortion care. Like the NAF Hotline, there are no published studies of the impact of either Planned Parenthood or TAF financial supports.

While there are significant sums of money channeled through national assistance funds, local and regional abortion assistance organizations also play an essential role in the ability of abortion seekers to access care. These nonprofit organizations, known as either abortion funds or practical support organizations, are collectively part of the National Network of Abortion Funds (NNAF), which lists over 100 local or regional abortion funds, up from 80 before *Dobbs*. In 2020, before the *Dobbs* decision, these local abortion funds collectively distributed approximately $10 million.^129^ In a recent news story, the NNAF Executive Director stated that the funds provided more than $36 million in abortion funding and $10 million in logistical support in 2023.^128^

Because of a greater commitment to telling their stories, local financial assistance funds have been the subject of a few research studies, providing valuable information about the users of local abortion financial assistance funds. A sophisticated analysis of the case management data for Access Reproductive Care (ARC) Southeast by Rice et al. offers a snapshot of how local abortion assistance funds fill the gaps left by the national abortion assistance funds.^130^ Over the 2017–2019 period, ARC Southeast served about 10,000 clients. Reflecting the demographics of both the South and abortion patients living on low incomes, approximately 80% of clients identified as non‐Hispanic Black, were publicly insured or uninsured, and had one or more children. None of these clients could use their public insurance (Medicaid) to cover the abortion. ARC Southeast made a median abortion fund contribution pledge of $75.

NNAF used to have a national financial assistance fund named after Dr. George Tiller, an all‐trimester abortion provider who was murdered by an anti‐abortion extremist in 2009. An analysis of this fund by Ely et al. provides additional information about people seeking abortion care after the first trimester of pregnancy.^42^ During the 2010–2015 period, the NNAF Tiller Memorial Fund provided assistance to approximately 4000 clients. Like the ARC Southeast fund, the majority of clients identified as African American and over half already had children. Because of its specialty nature, three‐quarters of the callers were beyond the first trimester of pregnancy and clients sought over $2000 in financial support; those aged 11–13 needed over $4000. The Tiller Memorial Fund contributed an average of $258 of support to these clients, but support ranged from $30 to $8000. Clients were able to contribute an average of $500 toward the price of their abortion.

An additional study by the same researchers examined the users of a Florida‐based local abortion assistance fund.^131^ From 2001 to 2015, the fund served 3216 clients, the majority of whom identified as people of color. On average, clients received $140 in financial support toward the price of their abortion care. However, the number of clients served increased over this period and the average amount of support correspondingly decreased. Over this period the average price of the abortion people were seeking increased and thus unmet financial need increased.

Leyser‐Whalen et al. have examined abortion funds serving clients on the US‐Mexico border, offering insight into the Latinx population's use of abortion assistance organizations. In the first study of the West Fund^132^ only 35% of callers were offered support for a median amount of $150 due to limited organizational resources. The study also found a greater need for and use of financial assistance by people later in pregnancy. In a second study of eight individuals who required abortion services in Texas at the time of Hurricane Harvey, the authors explored how intersecting oppressions, natural disasters, and interpersonal violence shaped the need for financial assistance to access abortion care.^132^

Drawing on applicant data collected from applications submitted to an abortion fund in Montana between 2013 and 2022, Garnsey, Liddell et al., sought to identify the thematic reasons why people need financial and emotional support.^133^, ^134^ When applicants apply, in addition to answering a range of demographic questions, representatives from the fund ask abortion seekers to write a brief description of their situation and reasons for funding. The descriptions served as the data for two separate analyses. The first paper^134^ examined the role of stigma in the need for seeking assistance, including perceived, enacted, and internalized stigma. Of note for the authors was applicants' emphasis on how stigma delayed access to care, increasing logistical and financial barriers and emotional distress. In the second paper,^133^ the authors connected relationship factors such as intimate partner violence, sexual violence, and the absence of a supportive partner to people's need for abortion funding. Unlike the other studies of abortion funds, the majority of those reporting race/ethnicity in this study identified as white. The authors' general conclusion for both studies was that in addition to reducing structural barriers to abortion care, abortion funds also provided emotional and social support to ease the harms of abortion stigmatization.

## WHAT THE ARTICLES IN THE VOLUME CONTRIBUTE TO OUR UNDERSTANDING

The contributions in this issue begin with a discussion of the importance of local abortion funds in helping people access abortion care. For their analysis, Smith et al.,^135^ go beyond simply describing the patient population and average amount of financial support to examine how users of the Kentucky Health Justice Network's (KHJN) Abortion Support Fund compare to the larger population of abortion patients as reported to the Kentucky Department for Public Health. They find that compared to the population characteristics reported to the state, KHJN supported a higher percentage of young people, people of color, and people at later gestations. This study therefore adds to the growing body of literature demonstrating higher need for financial support to obtain abortion care among those from structurally marginalized groups. Consistent with abortion funds studies by Rice et al.^130^ and Ely et al.^42^, ^131^ KHJN callers disproportionately identified as Black. Further, like Liddell et al.^133^ Smith et al. found high numbers of KHJN clients who identify as white, which may reflect the high rates of poverty among white Kentuckians. Other scholars of the Appalachian region of the US have documented the large need for improved access to reproductive health care.^97^, ^136^, ^137^, ^138^, ^139^

In their article, White et al.^140^ document how abortion fund staff and volunteers do much more than simply provide funds to cover the price of abortion care. Through in‐depth interviews with fund staff and volunteers, the authors explored respondents' experiences with callers whose appointments had been canceled or who traveled out of state to obtain abortion care following a March 2020 Texas state executive order that prohibited most abortions. They conclude that fund staff and volunteers provided a bridge between caller information gaps about the services and financial resources available, working proactively to help create plans to secure care that accounted for callers' specific needs. Of note was the emotional support provided so callers felt it was possible to overcome logistical hurdles to get an abortion, even if that required out‐of‐state travel. This study provides some of the first insight into how abortion funds adapted to address the growing demand for out‐of‐state care, which later accelerated post‐*Dobbs*. It also explores how multiple abortion funds need to work together as the abortion access landscape becomes more complex and adds to the literature on the emotional support role abortion fund staff and volunteers play when interacting with clients.^134^, ^141^, ^142^

Formal abortion funds are not the only mechanism for accessing financial support and, indeed, these funds also have to raise resources. In their research brief, Snyder and Grewal^143^ analyze crowdfunding activity on two platforms with differing ideological orientations in the US following the premature leak of the *Dobbs* decision. They examine campaigns for organizations providing abortion access or seeking to reduce abortion access, as well as individuals seeking abortion access and those with needs resulting from choosing not to access abortion care. They find that in a reversal from pre‐*Dobbs* crowdfunding,^144^, ^145^ abortion access campaigns tended to outperform other anti‐abortion‐related campaigns.

The next set of articles moves our attention from the discussion of privatized financing of abortion care to the critical role Medicaid continues to play and can play in the future. Updating the Guttmacher Institute's prior study of the characteristics of abortion patients,^48^ Jones compares patient characteristics and issues related to payment for abortion across patients residing in states where state Medicaid funds covered abortion and those operating under Hyde restrictions.^146^ She finds that 62% of respondents in Medicaid states used this method to pay for care, up from 51% in 2017. Like abortion funds, the demographics of Medicaid users for abortion reflect deep structural inequities, and over two‐thirds are Black or Latinx, of the lowest income group, and having a second‐trimester abortion. However, Medicaid alone is not enough to eliminate the need for supplemental support. Although two‐thirds of respondents in Hyde states had to raise money for abortion, 28% reported still needing to do so in Medicaid states. Jones ends the article by challenging states to eliminate state‐level prohibitions on Medicaid coverage.

In their article, Heil et al. examine the consequences of those policy decisions.^147^ With retrospective procedure and patient‐level data obtained from clinics, they assess changes in procedure volume and patients' share of total procedure price (patient price) following the decision in two states (Illinois and Maine) to expand state Medicaid to cover abortion and in one state (West Virginia) to eliminate that coverage. They find that Illinois and Maine extending state Medicaid coverage to abortion care contributed to an immediate overall increase in abortion access and a decrease in patient price after policy implementation as compared to pre‐implementation. Importantly, from a reproductive equity perspective, the authors find overall improved access specifically for people of color. Conversely, when West Virginia discontinued coverage, access to care decreased, patient price increased, and the share of abortion procedures among people of color decreased. This article provides support for Jackson and Rendall's finding that lifetime abortion incidence is higher when people have access to Medicaid coverage.^36^

Using qualitative methods, Quasebarth et al. help provide more context to implementing the Illinois policy change related to Medicaid abortion coverage.^148^ Between July 2021 and February 2022, the authors interviewed Illinois residents who recently sought abortion care in the first trimester of pregnancy. They also interviewed key informants with experience providing or billing for abortion or supporting insurance policy implementation in Illinois. Not surprisingly, given Heil et al.' findings, participants in this study found that individuals insured by Illinois Medicaid or eligible for enrollment received full financial coverage for their abortions. Participants who benefited from abortion coverage expressed relief, gave examples of other financial needs they could prioritize, and described feeling in control of their abortion experience. Many key informants echoed patients' sentiments about the value of Medicaid coverage. However, they spoke to the unique challenges associated with Illinois' use of managed care plans to facilitate Medicaid coverage. This was especially true for Hyde‐allowable abortions, which were billed differently through managed care than those not allowed by Hyde but paid for by the Illinois state Medicaid. Further, while informants welcomed Medicaid reimbursement rates, which had been raised as part of implementation, they also outlined some ongoing challenges, including additional paperwork, complications for patients with both Medicaid and Medicare, and concerns regarding delays in Medicaid reimbursement. This study suggests that many of the findings of prior work on the administrative challenges of accepting Medicaid for abortion care continue.^115^, ^116^, ^117^, ^118^, ^119^

Scanning back out to the larger abortion service delivery universe, Upadhyay et al. document the changing price of medication abortion services in the US.^149^ Project staff collected data by making calls to or examining the websites of all publicly advertising abortion facilities. From this, they are able to report that the national median price for medication abortion remained consistent at $568 in 2021 and $563 in 2023. However, medication abortions provided by virtual clinics had lower prices than in‐person care and this difference widened over time. The median cost of a medication abortion offered in person increased from $580 in 2021 to $600 by 2023, while the median price of a medication abortion offered by virtual clinics decreased from $239 in 2021 to $150 in 2023. Among virtual clinics, few (7%) accepted Medicaid. Median prices in states that accept Medicaid were generally higher than in states that did not.

Despite the overall lower advertised price of telemedicine medication abortion, the service is still priced too high for many abortion seekers. Foster et al. offer us the first insight into use of telemedicine medication abortion care through a Shield Law provider and the amount patients using that service actually pay for care.^150^ While the advertised price for the service was $250, patients were allowed to “pay what they can.” On average patients paid $134.50 with 29% paying less than $25. Patient comments about their care contained substantial attention to the financial hardship that both necessitated the abortion and made access difficult. These findings are consistent with prior studies documenting the differential between what people are able to pay for abortion care and the price of abortion, a gap historically filled by abortion funds.^42^, ^130^, ^131^ The efforts of Shield Law providers are one positive antidote to the horrendous assault on reproductive autonomy committed by the *Dobbs* decision.

While the experiences of individuals who are still able to obtain a desired abortion are a bright spot, the final two manuscripts in this special issue offer a reminder of what is at stake for all of society when abortion care is banned. We are excited for this special issue to include a reprinting of the amici curiae brief submitted by 150 economists in support of legal abortion in the *Dobbs* case.^151^ The brief challenges Mississippi's argument that there is no reason to believe that access to abortion impacts the ability of women to participate in the economic and social life of the nation. To do this, the economists, led by Myers and Srinivasan, summarize the existing literature on the effects of abortion access on birth rates with downstream social and economic effects including women's educational attainment and job opportunities. In addition to providing a review of existing literature, the amici curiae brief highlights for the Court how causal‐inference tools have advanced and been applied to isolate and measure the impacts of abortion legalization in the US and to model what would happen if *Roe* were overturned or limited. They find that women of every demographic group would be affected, and that reduced abortion access would have the greatest effect on young women and women of color. Readers not familiar with these statistical methods will find a user‐friendly guide to understanding their application to the question of the impact of abortion access on societies and economies.

In the final article in this issue, Speice leverages previous research and publicly available datasets to calculate abortion outcomes, individual costs, and public costs in three scenarios in which abortion care is banned in Ohio.^152^ In line with the findings summarized by Meyers and Srinivasan,^151^ Speice finds that the total additional economic impact of restricted abortion access in Ohio is significant, ranging between $98.8 million and $118.4 million but as high as $551.4 million per year. While these impacts have never come to fruition due to the passage of Ohio Amendment 1 in 2023, which enshrined the legal right to abortion until the point of fetal viability in Ohio's Constitution, the analysis here may apply to other states where legal restrictions have been implemented.

## CONCLUSIONS

Money matters in the context of abortion. This review demonstrates that a broad range of literature exists and that scholars are engaging with new questions, new populations, and new methods to expand what we know about the economics of abortion. It also reveals that there are many unexplored questions about how the business aspects of abortion care impact overall access and how changes in care utilization impact the sustainability of abortion providers. The importance of Medicaid to abortion access continues to be evident in research findings, demonstrating the imperative of eliminating the federal Hyde Amendment and making needed changes in state provider reimbursement policies and rates. The complex abortion assistance system that has developed to fill in for Medicaid system failures is strained in the aftermath of *Dobbs*, necessitating a reimagining of how abortion care is financed and supported in the US while ensuring that the emotional support needs of abortion seekers continue to be met.

## Endnotes

^1^

*Roe v. Wade* allowed states to prohibit abortion after the point of potential fetal viability, which is approximately the end of the second trimester. However, many factors impact viability, and thus a gestational age should not be specified.

^2^
I use the phrase "broadly legal" here because no state in the US has fully decriminalized abortion and only eight states do not have a timing‐based limits articulated in the law. Even within those states, other laws and regulations limit the provision of care and pregnant people can still be punished for taking particular actions with their pregnancies.

^3^
After the *Roe* decision in 1973, the organization changed its name to the National Abortion Rights Action League, then again in 1993 to the National Abortion and Reproductive Rights Action League and, in 2003 to NARAL Pro‐Choice America. In 2023, it formally resigned the NARAL acronym and adopted the new name Reproductive Freedom for All.

^4^
As the pro‐choice movement responded to reproductive justice activists' critiques, the social movement for abortion rights began to be identified as the reproductive health, rights, and justice movement.

^5^
State financial coverage should not be interpreted as a state government being supportive of abortion rights, as 10 states allow this care under a court order, and only nine cover abortion voluntarily.

^6^
Although Planned Parenthood is a significant healthcare system itself, it is not part of a more extensive system that generates revenues through inpatient or well‐reimbursed specialty care. While misunderstood by its opponents, providing other family planning services, whether paid for by Title X, Medicaid, or private insurance, is inadequate to cover the actual costs of providing those services. As such, financial losses for care provision at Planned Parenthood facilities are covered by donations (individual and institutional).

^7^
While closely aligned, the NAF Hotline is a separate entity from the organization known as NAF.
